# The sound of temporal epilepsy or maybe it’s just dissociation

**DOI:** 10.1192/j.eurpsy.2026.11550

**Published:** 2026-07-31

**Authors:** S. Kocijančič Azzaoui, A. Andlovic, J. Knific

**Affiliations:** 1 Psycho-oncology; 2 Neuro-oncology, Oncology institute, Ljubljana, Slovenia

## Abstract

**Introduction:**

Dissociative disorder is an uncommon, though not unheard-of, condition that presents with the loss or disturbance of normal motor, sensory, or cognitive functions. Patients may experience symptoms of derealization or depersonalization, which are characterized by feelings of detachment from oneself or reality, along with distorted perception. While it is often a diagnosis of exclusion, it can have neurological or physical causes.

**Objectives:**

This case report describes a 42-year-old male with a grade 2 astrocytoma who presented with headaches and episodes resembling absence seizures.

**Methods:**

Previous workup included the surgical resection of a left temporal lesion, histologically defined as MGMT promoter non-methylated and IDH-mutated astrocytoma. The patient subsequently underwent chemotherapy and radiation therapy, with disease stability since November 2022. He discontinued antiepileptic medication in 2023 due to the absence of seizures. In 2025, he began experiencing episodes of impaired awareness (i.e., “absences”) lasting up to 30 seconds, prompting referral from the oncologist to a neurologist. Neurological examination revealed no significant abnormalities, except for mild pronation of the right arm. An EEG performed in May 2025 after sleep deprivation showed slowed activity in the left temporal region, corresponding to the postoperative area, but no epileptiform discharges.

**Results:**

Since the EEG and the neurological examination did not reveal pathological findings, the neurologist referred the patient to a psychiatrist. At that time, the patient was taking lacosamide 50 mg twice daily but reported no reduction in the frequency or duration of absences. He described the episodes as brief states of detachment from reality, accompanied by emotional numbness and loss of attention, occurring several times per day, without clear triggers. He also reported recurrent headaches, resistant to paracetamol. His primary physician prescribed duloxetine for headache, depressive, and anxiety symptoms. At a dose of 60 mg, the patient experienced improved focus and energy, but no change in seizure-like episodes. At his most recent follow-up, the duloxetine dosage was increased. The patient described his episodes in more detail—as brief auditory phenomena, such as hearing familiar melodies or unrecognizable tunes, without external stimuli.

**Conclusions:**

Dissociative disorder remains a diagnosis of exclusion. In this case, however, the patient’s history of left temporal astrocytoma could also explain the symptoms. A repeat EEG may help clarify whether these episodes represent temporal lobe seizures rather than dissociative (non-epileptic) episodes. Continuous clinical follow-up and multimodal assessment are warranted to distinguish between neurological and psychogenic causes, especially since IDH mutation has emerged as an independent risk factor for the development of seizures in glioma patients.

**Disclosure of Interest:**

None Declared

